# Cancer-associated fibroblasts and innate immune cells: orchestrators of the immunosuppressive tumor microenvironment

**DOI:** 10.3389/fimmu.2026.1876751

**Published:** 2026-10-19

**Authors:** Dmitry A. Zinovkin, Yulia S. Korneva, Heba Mohammed Arafat, Ohood Mohammed Shamallakh, Ekaterina S. Zinovkina, Denis A. Davydov, Anna S. Portyanko, Irina I. Slepokurova, Yulia A. Lyzikova, Md Zahidul Islam Pranjol

**Affiliations:** 1 Department of Pathology, Gomel State Medical University, Gomel, Belarus; 2 Department of Prevention and Treatment of Wound Infections, National Medical Research Center of Traumatology and Orthopedics named after R.R. Vreden, St. Petersburg, Russia; 3 Department of Pathologycal Anatomy, North-Western State Medical University named after I.I. Mechnikov, St. Petersburg, Russia; 4 Department of Chemical Pathology, School of Medical Sciences, Health Campus, Universiti Sains Malaysia, Kubang Kerian, Kelantan, Malaysia; 5 Department of Medical Laboratory Sciences, Faculty of Health Sciences, Islamic University of Gaza, Gaza City, Palestine; 6 Department of Radiology and Radiotherapy, Gomel State Medical University, Gomel, Belarus; 7 National Molecular Genetics Laboratory of Cancer Research, N.N. Alexandrov National Cancer Center of Belarus, Minsk, Belarus; 8 Department of Obstetrics and Gynecology, Gomel State Medical University, Gomel, Belarus; 9 School of Life Sciences, University of Sussex, Brighton, United Kingdom

**Keywords:** cancer-associated fibroblasts, cytokines, immune suppression, immunotherapy, innate immunity, tumor microenvironment, tumor-associated macrophages, tumor-associated neutrophils

## Abstract

The tumor microenvironment (TME) represents a complex, dynamic, and heterogeneous system in which stromal and immune elements interact to influence tumor development and mechanisms of immune escape. Within the stromal compartment, cancer-associated fibroblasts (CAFs) have been identified as key mediators of immune regulation in the TME. Emerging evidence indicates that CAFs engage in extensive crosstalk with innate immune cell populations—including myeloid-derived suppressor cells (MDSCs), tumor-associated macrophages (TAMs), tumor-associated neutrophils (TANs), natural killer (NK) cells, mast cells, and dendritic cells (DCs). Through these interactions, CAFs contribute to the establishment and maintenance of an immunosuppressive milieu that supports tumor progression and facilitates immune evasion. This review summarizes the molecular interactions between CAFs and innate immune cells in the TME. It highlights how CAF-derived factors such as IL-6, TGF-β, CXCL12, and CSF-1 regulate immune cell recruitment and polarization, promoting immunosuppression, tumor progression, angiogenesis, and metastasis. Additionally, feedback signals from immune cells further sustain CAF activation, creating a self-reinforcing immunosuppressive microenvironment. Elucidating these multidirectional interactions offers important insights into tumor biology and identifies promising therapeutic avenues. Targeting CAF-associated signaling pathways or interfering with CAF–immune cell communication may help restore anti-tumor immune responses and improve the effectiveness of cancer immunotherapy strategies.

## 1 Introduction

The tumor microenvironment (TME) is a complex and dynamic system composed of cancer cells, stromal cells, and immune cells, all of which interact to influence tumor growth, immune evasion, and metastasis (1). Among the stromal components, cancer-associated fibroblasts (CAFs) play a particularly important role. They are not merely passive elements within the tumor but actively contribute to its development through interactions with cancer cells and the immune system (2, 3). In particular, their communication with innate immune cells—such as myeloid-derived suppressor cells (MDSC), tumor-associated macrophages (TAMs), tumor-associated neutrophils (TANs), natural killer (NK) cells, mast cells, and dendritic cells (DCs) – plays a crucial role in shaping an immunosuppressive and tumor-promoting environment (4, 5).

Innate immune cells detect and eliminate malignant cells as the first line of immune defense. Within the TME, CAFs alter their function, shifting them from tumor-suppressive to tumor-promoting roles. This reprogramming occurs through the secretion of cytokines, chemokines, and growth factors that recruit, activate, and polarize innate immune cells into pro-tumorigenic phenotypes (6). In many, although not all, tumor types, CAFs induce the differentiation of monocytes into immunosuppressive M2 TAMs, drive the polarization of TANs toward an N2 phenotype that enhances angiogenesis and metastasis, and reduce NK-cell activity. CAFs interact with mast cells and DCs, suppressing anti-tumor immunity and facilitating immune evasion (7).

These interactions are not unidirectional; innate immune cells reciprocally influence CAF behavior, creating a self-reinforcing loop that sustains tumor progression. For instance, M2 macrophages and activated neutrophils can enhance CAF activation and extracellular matrix (ECM) remodeling, while mast cells and DCs contribute to stromal remodeling and immune suppression under the influence of CAF-derived signals (8, 9).

A deep understanding of the interactions between CAFs and innate immune cells is essential for the development of effective therapeutic strategies. Targeting these interactions offers the potential to disrupt the immunosuppressive TME, restore anti-tumor immunity, and improve patient outcomes (10). This review explores the multifaceted roles of CAFs in modulating innate immune cell behavior within the TME, highlighting the mechanisms underlying these interactions and their implications for cancer therapy. Investigating these complex interactions will facilitate the development of novel therapeutic approaches capable of overcoming the barriers of the tumor microenvironment and improving cancer treatment outcomes (11, 12).

## 2 Interactions between CAFs and innate immune cells in the tumor microenvironment

CAFs critically shape innate immunity within the tumor microenvironment by recruiting and reprogramming myeloid-derived suppressor cells, tumor-associated macrophages, neutrophils, mast cells, NK cells, and dendritic cells (**Figure 1**).

**Figure 1:**
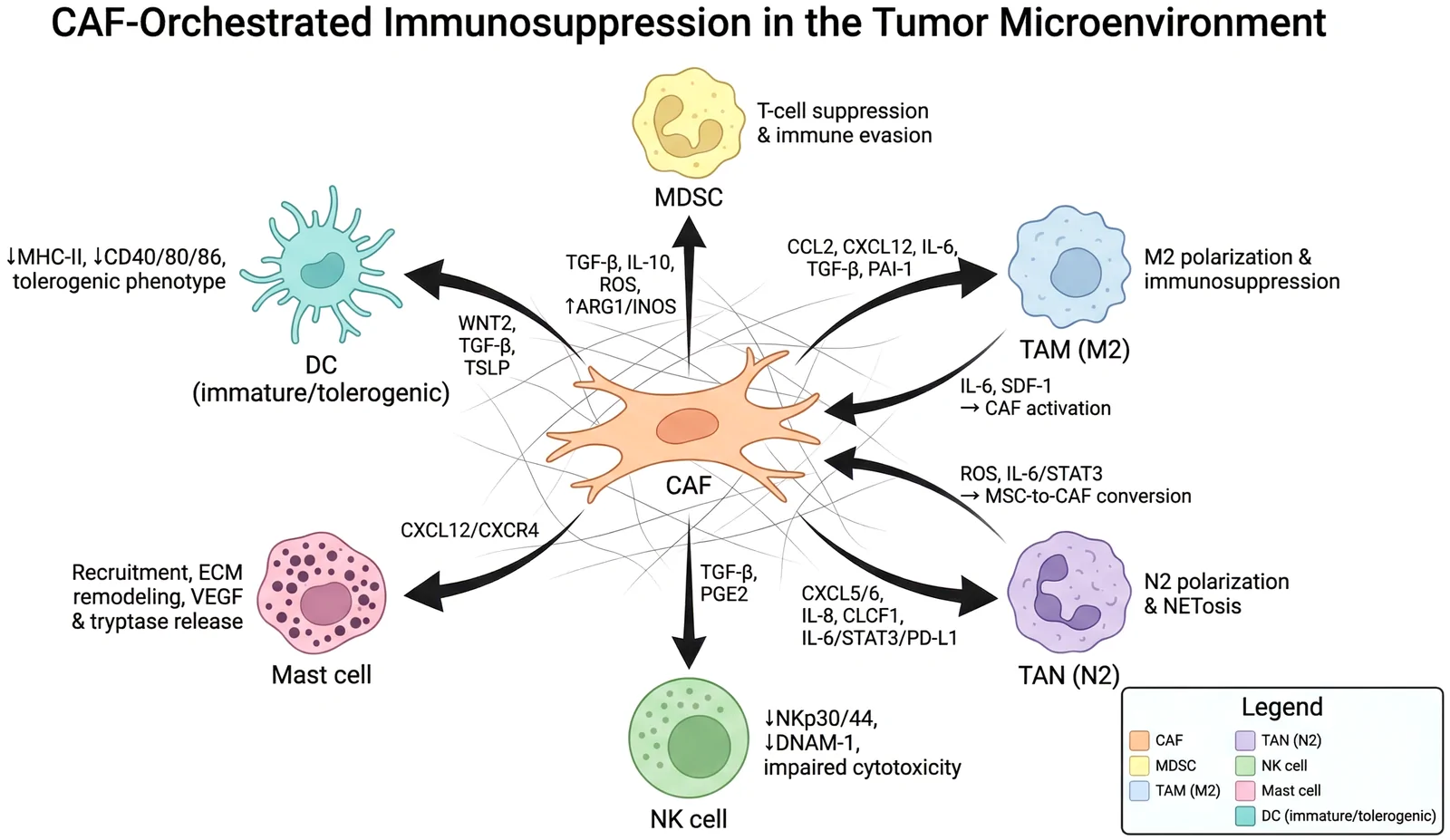
CAF-orchestrated immunosuppression in the tumor microenvironment. Schematic illustration of the multifaceted crosstalk between Cancer-Associated Fibroblasts (CAFs) and distinct immune cell populations that collectively drive an immunosuppressive tumor microenvironment. CAFs secrete a diverse array of cytokines, chemokines, and metabolic mediators to modulate immune cell recruitment, polarization, and function. Specifically, CAFs enhance Myeloid-Derived Suppressor Cell (MDSC)-mediated T-cell suppression and immune evasion through the secretion of TGF-β, IL-10, and ROS, alongside the upregulation of ARG1 and iNOS. CAFs promote the polarization of macrophages toward an immunosuppressive M2 phenotype (TAMs) via CCL2, CXCL12, IL-6, TGF-β, and PAI-1, establishing a reciprocal loop where TAM-derived IL-6 and SDF-1 further activate CAFs. Similarly, CAFs drive neutrophil polarization toward an N2 phenotype (TANs) and induce NETosis through CXCL5/6, IL-8, CLCF1, and IL-6/STAT3/PD-L1 signaling, while TAN-derived ROS and IL-6/STAT3 facilitate the conversion of Mesenchymal Stem Cells (MSCs) into CAFs. Furthermore, CAFs induce a tolerogenic, immature phenotype in Dendritic Cells (DCs) characterized by downregulated MHC-II and co-stimulatory molecules (CD40/80/86) via WNT2, TGF-β, and TSLP secretion. In the innate lymphoid compartment, CAF-derived TGF-β and PGE2 impair Natural Killer (NK) cell cytotoxicity and downregulate the expression of activating receptors NKp30/44 and DNAM-1. Finally, through the CXCL12/CXCR4 axis, CAFs recruit mast cells, promoting extracellular matrix (ECM) remodeling and the release of VEGF and tryptase. Collectively, these interactions inhibit anti-tumor immunity and support tumor progression.

### 2.1 CAF heterogeneity and single-cell transcriptomics redefine stromal regulation of innate immunity

The traditional view of CAFs as a relatively homogeneous stromal population has been fundamentally revised by recent advances in single-cell RNA sequencing (scRNA-seq), spatial transcriptomics, and multi-omics technologies. These approaches have demonstrated that CAFs comprise multiple phenotypically and functionally distinct subpopulations exhibiting remarkable lineage plasticity and spatial specialization within the TME (13). Rather than representing fixed cellular identities, CAF phenotypes exist along a dynamic continuum shaped by reciprocal interactions with malignant cells, immune cells, ECM, and local biochemical cues. Consequently, CAF heterogeneity has emerged as a critical determinant of tumor progression, immune regulation, metastatic dissemination, and therapeutic response (14).

The concept of CAF heterogeneity was initially established by Öhlund et al., who identified two major fibroblast populations in pancreatic ductal adenocarcinoma: myofibroblastic CAFs (myCAFs), characterized by high expression of ACTA2 (α-SMA) and extracellular matrix-related genes, and inflammatory CAFs (iCAFs), distinguished by robust secretion of inflammatory cytokines and chemokines including IL-6, IL-8 (CXCL8), CXCL1, CXCL2, CXCL12, and leukemia inhibitory factor (LIF) (15, 16). Subsequent studies by Biffi et al. demonstrated that these phenotypes are regulated through antagonistic signaling pathways, where IL-1-induced JAK/STAT activation promotes the inflammatory iCAF phenotype, whereas TGF-β signaling suppresses inflammatory transcriptional programs and drives differentiation toward contractile myCAFs (17, 18). Importantly, these populations remain highly plastic and may interconvert in response to alterations in cytokine gradients, hypoxia, mechanical stress, and therapeutic interventions, highlighting that CAF subsets represent functional states rather than permanently differentiated cell types (19).

The discovery of antigen-presenting CAFs (apCAFs) by Elyada et al. further expanded the functional complexity of stromal fibroblasts. Unlike classical fibroblasts, apCAFs express major histocompatibility complex class II (MHC II) molecules together with CD74, enabling antigen processing and presentation. However, these cells lack essential co-stimulatory molecules required for effective T-cell activation, suggesting that antigen presentation by apCAFs contributes primarily to immune tolerance rather than productive adaptive immunity (20, 21). Recent studies further indicate that apCAFs influence dendritic cell maturation and indirectly modulate macrophage polarization, thereby linking stromal remodeling with both innate and adaptive immune suppression (22).

Recent pan-cancer single-cell and spatial transcriptomic analyses have considerably broadened the CAF classification beyond the original myCAF/iCAF/apCAF framework. Multiple additional subsets have now been identified across solid tumors, including vascular-associated CAFs (vCAFs), matrix-producing CAFs (mCAFs), proliferating CAFs (pCAFs), interferon-responsive CAFs (ifnCAFs), metabolic CAFs (meCAFs), and wound-healing or developmental CAF states (23). Rather than being universally conserved, these subsets display pronounced tissue-specific transcriptional programs while maintaining several conserved signaling modules involved in extracellular matrix remodeling, inflammatory regulation, angiogenesis, and immune modulation. Spatial transcriptomics has further demonstrated that CAF subsets occupy distinct anatomical niches within tumors (24). MyCAFs are typically located adjacent to malignant epithelial nests where they generate dense collagen-rich ECM and elevated tissue stiffness, whereas iCAFs preferentially localize within inflammatory stromal regions enriched with immune infiltrates (20). Conversely, vCAFs are closely associated with tumor vasculature and actively regulate endothelial cell function, angiogenesis, and vascular remodeling (25). This spatial compartmentalization provides an additional layer of functional specialization that cannot be resolved by conventional bulk transcriptomic analyses (26).

Emerging multi-omics approaches integrating transcriptomics, chromatin accessibility, epigenetic profiling, proteomics, metabolomics, and spatial imaging have further demonstrated that CAF heterogeneity reflects continuous transcriptional adaptation rather than discrete lineage commitment (27). These studies suggest that fibroblasts originating from resident tissue fibroblasts, mesenchymal stem cells, pericytes, adipocytes, endothelial cells, or epithelial cells undergoing epithelial-to-mesenchymal transition may converge toward overlapping CAF phenotypes under the influence of TGF-β, IL-1, hypoxia, mechanical signaling, and metabolic stress (28). Such plasticity explains the remarkable functional diversity observed across different tumor types and disease stages.

Distinct CAF subsets exhibit preferential interactions with specific innate immune-cell populations rather than exerting uniform immunoregulatory effects. iCAFs predominantly regulate MDSCs, M2-like TAMs, and tumor-associated neutrophils through IL-6, CXCL12, CXCL5, and G-CSF, whereas myCAFs influence macrophage function indirectly via extracellular matrix remodeling and tissue stiffening (29). Antigen-presenting apCAFs primarily modulate dendritic-cell maturation and immune tolerance through MHC II-dependent mechanisms, while vCAFs regulate endothelial activation, angiogenesis, and immune-cell trafficking within perivascular niches (30).

The evidence supporting these subtype-specific interactions derives from complementary experimental systems. CAF taxonomy has largely emerged from murine lineage-tracing and single-cell transcriptomic studies, whereas their spatial organization and clinical relevance have been confirmed in human single-cell and spatial transcriptomic datasets (31). In contrast, many mechanistic CAF–immune interactions have been established primarily in *in vitro* co-culture models and subsequently validated in selected animal models, highlighting the need for caution when extrapolating findings across different tumor types (32).

Importantly, increasing evidence indicates that distinct CAF subsets differentially regulate innate immune responses. Inflammatory CAFs represent the major source of IL-6, CXCL8, CXCL12, CCL2, CXCL1, CXCL5, and granulocyte colony-stimulating factor (G-CSF), thereby orchestrating the recruitment and expansion of immunosuppressive myeloid populations (33). Through activation of the CXCL12/CXCR4 axis, iCAFs promote the accumulation of TAMs, myeloid-derived suppressor cells (MDSCs), and neutrophils while simultaneously restricting cytotoxic lymphocyte infiltration into tumor nests. IL-6-mediated activation of the STAT3 pathway further enhances M2-like macrophage polarization, promotes suppressive myeloid differentiation, and supports tumor progression (34). Similarly, CXCL8 produced predominantly by inflammatory CAFs stimulates neutrophil recruitment and favors the development of pro-tumorigenic neutrophil phenotypes characterized by increased angiogenic and immunosuppressive activity (35).

In contrast, myCAFs primarily regulate immune responses indirectly through extracellular matrix remodeling and mechanical alterations of the tumor stroma. Excessive deposition of collagen, fibronectin, periostin, and hyaluronan generates a dense fibrotic barrier that limits immune cell trafficking and reduces penetration of therapeutic agents (36). Matrix remodeling also influences macrophage activation through integrin-mediated mechanotransduction while facilitating tumor invasion and angiogenesis (37). Furthermore, vascular CAFs actively participate in endothelial cell activation and vascular remodeling by secreting VEGF, PDGF, angiopoietins, and matrix metalloproteinases, thereby coordinating angiogenesis with immune cell trafficking and tumor dissemination (38).

Recent studies additionally demonstrate that several CAF subsets directly suppress NK cell function. CAF-derived TGF-β, prostaglandin E2, CXCL12, and extracellular matrix proteins reduce NK-cell cytotoxicity, impair interferon-γ production, and decrease expression of activating receptors including NKG2D (39). CAF-derived PD-L1, together with secretion of IL-6 and transforming growth factor-β, further contributes to immune exhaustion and resistance to immune checkpoint blockade (40). Collectively, these mechanisms establish CAFs as central regulators of innate immune suppression rather than passive structural components of the tumor stroma (41).

The rapidly evolving understanding of CAF biology therefore indicates that therapeutic strategies should no longer consider CAFs as a single targetable population. Broad depletion of fibroblasts has produced inconsistent clinical outcomes, likely reflecting the coexistence of tumor-promoting and potentially tumor-restraining CAF subsets (42). Current evidence instead supports precision stromal targeting based on subtype-specific vulnerabilities, including inhibition of IL-1/JAK-STAT signaling to suppress inflammatory CAF programs, blockade of TGF-β-mediated myofibroblast activation, disruption of the CXCL12/CXCR4 axis, modulation of PD-L1-dependent immune suppression, and selective targeting of vascular or matrix-associated CAF populations (43). As single-cell and spatial multi-omics technologies continue to refine CAF taxonomy, integrating stromal heterogeneity into models of tumor immunity will be essential for understanding innate immune regulation and for developing more effective combination immunotherapies (44).

Importantly, much of the current understanding of CAF heterogeneity has been generated using single-cell transcriptomic datasets and lineage-tracing experiments in murine models. Although similar CAF subsets have subsequently been identified in multiple human malignancies, the nomenclature, biological functions, and therapeutic relevance of these populations remain incompletely standardized across tumor types. Therefore, caution is warranted when extrapolating subtype-specific findings between different cancers.

### 2.2 Myeloid-derived suppressor cells and CAFs interactions

MDSCs are a heterogeneous population of immature myeloid cells that expand under pathological conditions, particularly within the TME, where they serve as critical mediators of immune evasion and tumor progression (45). Unlike mature myeloid cells that contribute to host defense, MDSCs fail to complete terminal differentiation and instead acquire potent immunosuppressive properties (46). In humans, these cells are primarily classified into two subsets: polymorphonuclear MDSCs (PMN-MDSCs) and monocytic MDSCs (M-MDSCs) (47). While PMN-MDSCs often constitute the majority of the MDSC population in solid tumors, M-MDSCs frequently exhibit higher suppressive activity on a per-cell basis and can further differentiate into TAMs (48).

The primary hallmark of MDSCs is their ability to orchestrate a multifaceted immunosuppressive network that disrupts both innate and adaptive immunity (49). One of the most well-characterized mechanisms involves the depletion of essential nutrients, such as L-arginine, through the high expression of arginase-1 (ARG1) and inducible nitric oxide synthase (iNOS) (50). This depletion leads to the downregulation of the T-cell receptor (TCR) ζ-chain and subsequent T-cell cycle arrest. Furthermore, MDSCs generate high levels of reactive oxygen species (ROS) and peroxynitrite, which cause TCR nitration and impair the ability of T-cells to recognize tumor antigens (51). Beyond these metabolic pathways, MDSCs contribute to the formation of a privileged niche for tumor growth by secreting inhibitory cytokines like TGF-β and IL-10, which promote the expansion of regulatory T-cells (Tregs) while inhibiting the cytotoxic activity of NK cells (52).

In addition to their immunological roles, MDSCs actively participate in the non-immunological remodeling of the TME. They are pivotal in stimulating angiogenesis through the secretion of pro-angiogenic factors, including vascular endothelial growth factor (VEGF) and matrix metalloproteinases (MMPs) (53). Moreover, MDSCs are implicated in the induction of the epithelial-to-mesenchymal transition (EMT), thereby promoting tumor dissemination and metastasis (54). Given their strong correlation with poor patient prognosis, MDSCs have emerged as high-priority therapeutic targets. Current strategies focus on MDSC checkpoint blockade and inhibiting their suppressive functions to restore anti-tumor immunity (55).

Beyond their intrinsic immunosuppressive properties, MDSCs are extensively regulated by CAF-derived soluble mediators and extracellular matrix remodeling, making CAFs one of the principal stromal orchestrators of MDSC accumulation within the tumor microenvironment (56). iCAFs represent a major source of CXCL12, CCL2, CXCL5, G-CSF, IL-6, and TGF-β, thereby establishing a chemokine-rich niche that recruits immature myeloid cells and prevents their terminal differentiation (57). Activation of the CXCL12/CXCR4 and CCL2/CCR2 signaling axes promotes continuous recruitment of monocytic and polymorphonuclear MDSCs, while IL-6-mediated STAT3 activation enhances ARG1, iNOS, and ROS production, reinforcing the suppressive phenotype of infiltrating MDSCs (58).

Additionally to soluble cytokines, myCAFs regulate MDSC behavior through extracellular matrix remodeling. Collagen-rich desmoplastic stroma, fibronectin, hyaluronan, and periostin create biomechanical niches that retain MDSCs within tumors through integrin-dependent adhesion and mechanotransduction, thereby prolonging their survival and immunosuppressive activity (59, 60).

The interaction is bidirectional: MDSCs reciprocally sustain CAF activation by secreting TGF-β, IL-1β, and matrix metalloproteinases, creating a positive feedback loop that promotes chronic stromal inflammation, angiogenesis, and resistance to immunotherapy. These findings identify the CAF–MDSC axis as a promising target for combination stromal-immunotherapeutic strategies.

### 2.3 Tumor-promoting interactions between cancer-associated fibroblasts and tumor-associated macrophages

The tumor stroma plays a fundamental role in cancer advancement and metastatic progression. It consists of diverse non-malignant cells, extracellular matrix (ECM) elements, and signaling molecules that interact with tumor cells, establishing a microenvironment that fosters tumor proliferation, invasion, and spread (61). Among the essential stromal components, CAFs and TAMs are particularly impactful in molding the TME and accelerating cancer progression. CAFs facilitate tumor growth by remodeling the ECM, releasing growth factors, modulating immune responses, and providing metabolic support (62). TAMs, which make up 30–50% of stromal cells in many cancers, exhibit functional plasticity and are categorized into two main subtypes: M1 macrophages, which have anti-tumor and pro-inflammatory roles, and M2 macrophages, which promote tumor growth, angiogenesis, and immune suppression. Together, CAFs and TAMs create a supportive environment that enables tumor cells to evade immune surveillance, acquire invasive properties, and metastasize to distant sites. Targeting these stromal components offers promising therapeutic strategies to complement traditional cancer treatments and improve patient outcomes (63).

Although TAMs are traditionally categorized into pro-inflammatory M1 and immunosuppressive M2 phenotypes, this binary classification is now recognized as an oversimplified conceptual framework rather than a representation of discrete macrophage populations (64). Recent single-cell RNA sequencing and spatial transcriptomic studies demonstrate that TAMs occupy a continuous spectrum of transcriptional and metabolic states, simultaneously expressing markers associated with both classical and alternative activation depending on local cytokine gradients, hypoxia, extracellular matrix composition, and tumor stage (65).

Accordingly, CAF-derived IL-6, CSF-1, CXCL12, CCL2, TGF-β, and IL-33 should be viewed as drivers of multiple immunoregulatory macrophage states rather than uniform inducers of a single M2 phenotype (66). Throughout this review, the term “M2-like TAMs” refers to functionally immunosuppressive macrophages while acknowledging their substantial biological heterogeneity across different tumor types.

Numerous studies have demonstrated that CAFs can promote the differentiation of human THP-1 monocytes into immunosuppressive M2 macrophages. It has been demonstrated that CAFs stimulate the production of reactive oxygen species (ROS) in THP-1 monocytes, which play a critical role in facilitating the polarization of M2 macrophages (67). CAFs attract monocytes to the tumor microenvironment predominantly via the CCL2-CCR2 signaling cascade (68), with additional modulation by cytokines such as SDF-1 and MCP-1 (69). After infiltrating the tumor site, monocytes may differentiate into M2-like TAMs under CAF-derived signals in several tumor models, acquiring immunosuppressive properties through the PD-1 signaling axis. The polarization of monocytes into M2 TAMs within the tumor microenvironment is significantly influenced by cytokines produced by CAFs, including IL-8, IL-33, IL-10, and TGF-β (70, 71). Zhou X. et al. (2022) demonstrated that CAFs in the tumor microenvironment can regulate the size of the TAM population by secreting CSF-1 (72). CAF-derived IL-6 has been reported to promote the development of immunosuppression and to stimulate tumor growth (72). Both CAFs and TAMs secrete PAI-1, a serine protease inhibitor, while CAFs further enhance this process by secreting CXCL12. Elevated PAI-1 levels reduce E-cadherin expression and increase vimentin production, facilitating EMT and subsequent metastasis (73). The study by Czekay R. et al. (2023) demonstrated that CAF-derived PAI-1 contributes to macrophage polarization toward the M2 phenotype and enhances tumor invasion via the LRP1/PI3K/AKT pathway. Additionally, CAF-driven M2 TAM activation alters the function of immunosuppressive T-regulatory cells in the tumor microenvironment (74).

Conversely, M2 TAMs also play a role in regulating CAF activation and tumor progression. In a study by Comito G. et al. (2013), it was shown that while CAFs promote TAM activity, M2-type macrophages can reciprocally enhance EMT and stimulate CAF activation by secreting soluble factors such as IL-6 and SDF-1 (75, 76).

Current research continues to elucidate novel aspects of the interaction between CAFs and TAMs within the tumor microenvironment, demonstrating their intricate and interdependent roles in sustaining tumor growth and progression. It should be noted, however, that much of this evidence derives from *in vitro* co-culture systems and murine models, and that the relative contribution of individual mediators varies considerably between tumor types. Due to their multifaceted pro-tumorigenic activity, both CAFs and TAMs are increasingly recognized as promising targets for the development of novel anti-cancer therapeutic strategies.

### 2.4 The CAF-TAN axis: the role of tumor-associated neutrophils in the CAF-mediated remodeling of the tumor microenvironment

TANs serve a paradoxical function in cancer progression, exhibiting both tumor-promoting and tumor-suppressing effects. Their specific role is influenced by factors such as the histopathological characteristics of the malignancy, the TME, and immunological conditions (67). Within the TME, neutrophils undergo polarization into either the N1 phenotype, which is anti-tumorigenic, or the N2 phenotype, which supports tumor advancement. N1 neutrophils are characterized by their maturity, brief lifespan, potent cytotoxicity, and robust capacity to elicit immune responses. Conversely, N2 neutrophils display an immature phenotype, exhibit diminished cytotoxic potential, and persist for longer periods, all while facilitating angiogenesis, metastasis, and immune suppression (6).

CAFs can recruit neutrophils and modulate their phenotype through multiple cytokine pathways, for example by secreting CXCL12 or by engaging CXCR2, although the resulting neutrophil states differ substantially among tumor types. The survival, activation, and functional modulation of TANs under CAF influence are mediated through the IL-6/STAT3-PD-L1 signaling pathway (77). More specifically, CAFs sustain neutrophil activity and longevity by engaging the IL-6/STAT3/programmed death ligand 1 (PD-L1) axis. When TANs are co-cultured with CAFs, they exhibit an upregulation of PD-L1, IL-8, CCL2, and TNF-α. These CAF-modulated neutrophils subsequently compromise T-cell immune functionality by manipulating the STAT3-PD-L1 pathway (78). Furthermore, CAF-derived cardiotrophin-like cytokine factor 1 (CLCF1) drives neutrophil differentiation toward the N2 phenotype, a process that exacerbates hepatocellular carcinoma (HCC) progression. Within the HCC microenvironment, IL-6 secreted by CAFs undermines T-cell activity and fosters immune tolerance by triggering the JAK-STAT3 signaling pathway in TANs (79).

Additionally, CAFs have been reported to enhance NETosis in several experimental models, whereby neutrophils generate extracellular traps, through the secretion of cytokines such as CXCL5, CXCL6, and IL-8. These molecular signals reshape TAN phenotypes and amplify NETosis (80). The release of neutrophil extracellular traps (NETs) induced by CAFs is further regulated by amyloid β, which CAFs secrete to influence NETosis through CD11b in a ROS-dependent manner. This phenomenon extends beyond the tumor microenvironment, impacting both systemic circulation and bone marrow activity (81).

The relationship between CAFs and neutrophils is bidirectional. Neutrophils, as significant generators of reactive oxygen species (ROS), have the capacity to transform mesenchymal stem cells (MSCs) into CAFs that express high levels of fibroblast activation protein (FAP). This transformation enhances the metastatic potential of gastric cancer cells via the IL-6/STAT3 signaling axis. In hepatocellular carcinoma, α-SMA+ CAFs secrete IL-6, which facilitates neutrophil recruitment and activates the STAT3 and c-Jun kinase-PD-L1 pathway. This signaling cascade not only triggers neutrophil apoptosis but also intensifies tumor progression (82).

The current evidence linking CAFs to NETosis should be interpreted according to the experimental model from which it originates. Most mechanistic studies demonstrating direct induction of neutrophil extracellular traps have been performed in *in vitro* co-culture systems, where CAF-derived IL-8, CXCL5, CXCL6, and inflammatory cytokines stimulate ROS-dependent NET formation (83). These findings have subsequently been supported in several murine tumor models, including hepatocellular carcinoma and gastric cancer, in which CAF-induced NETosis promotes metastatic dissemination and immune suppression. In contrast, evidence from human tumors remains predominantly correlative (84). Spatial analysis of human non-small cell lung cancer biopsies has identified activated CAFs in close proximity to NET structures; however, the observational nature of these findings precludes conclusions about a direct causal relationship between CAF activation and NET formation (85).

Importantly, CAF–NET interactions appear to be highly context-dependent. Differences in CAF transcriptional programs, neutrophil phenotypes, and cytokine landscapes across tumor types suggest that the relative contribution of IL-8, CXCL5, TGF-β, CLCF1, and other mediators varies considerably between malignancies. Therefore, NETosis should be regarded as a dynamic stromal–immune mechanism whose biological significance depends on the molecular and anatomical characteristics of individual tumors.

### 2.5 Natural killer cells in the immunosuppressive tumor microenvironment: modulation by CAFs

NK cells serve as pivotal effectors in anti-tumor immunity within the TME. Most available evidence derives from co-culture systems and selected solid tumor models. A comprehensive review by Chan et al. describes a spectrum of activating NK-cell receptors, including DNAM-1, LFA-1, NKp30, NKp44, NKp46, and NKG2D, the ligands of which are present on the surface of target cells, including malignant cells. Notably, indoleamine 2,3-dioxygenase (IDO), an immunosuppressive enzyme secreted by various cancer cells, plays a critical role in dampening NK cell activity, thereby facilitating immune evasion within the TME (86).

Upon activation, NK cells engage in cytotoxic responses through degranulation, releasing perforin and granzyme B. Perforin mediates the formation of transmembrane pores, allowing granzyme B to infiltrate target cells and trigger caspase-dependent apoptotic pathways. Furthermore, NK cells express members of the TNF superfamily, such as Fas ligand (FasL) and TNF-related apoptosis-inducing ligand (TRAIL), which induce apoptosis through extrinsic signaling pathways upon direct cell contact (87).

Within co-culture models, CAFs have been demonstrated to suppress key mechanisms governing NK cell activation. Specifically, CAFs inhibit IL-2-induced upregulation of activating receptors such as NKp44, NKp30, and DNAM-1, while simultaneously impairing the acquisition of cytolytic granules. DNAM-1 downregulation necessitates direct cell-cell interaction with CAFs, whereas the suppression of NKp44 and NKp30 expression is mediated by prostaglandin E2 (PGE2) secreted by CAFs. Additionally, podoplanin-expressing fibroblast subpopulations interfere with CCL21/CCR7-mediated adhesion and NK cell migration within the TME, further reinforcing their immunosuppressive function (88).

TGF-β signaling is a well-documented mechanism through which NK cell cytotoxicity is curtailed. Studies have elucidated that TGF-β impairs NK cell function via multiple pathways, including epigenetic modifications and upregulation of the mTOR signaling cascade. Moreover, TGF-β inhibits interferon-γ (IFNγ) secretion in a SMAD3-dependent manner, thereby attenuating the anti-tumor response of CD4+ TH1 cells. α-SMA-positive CAFs constitute a primary source of TGF-β within the TME, further contributing to its immunosuppressive landscape by modulating NK cell activity (89).

### 2.6 Mast cells and CAFs: a synergistic partnership in tumor progression

Mast cells are tissue-resident immune cells that respond to local environmental stimuli by releasing a variety of chemical mediators through specific signaling pathways. These mediators play a dual role, modulating protective immune responses against pathogens like bacteria, parasites, and viruses, while also contributing to allergic diseases and cancer progression. Mast cells are known to infiltrate many solid tumors, where they are referred to as tumor-associated mast cells (TAMCs) (90).

Mast cells exhibit distinct polarization states. Type 1 mast cells are cytotoxic, producing granzyme B, IL-9, and histamine, which promote dendritic cell maturation and, in murine models, inhibit tumor growth. Conversely, type 2 mast cells secrete pro-angiogenic and pro-metastatic factors such as VEGF, TGF-β, IL-13, FGF, and MMP-9 (91).

TAMCs, which can have both tumor-suppressing and tumor-promoting effects, are recruited to the tumor microenvironment by CAFs via the CXCL12/CXCR4 axis. *In vitro* studies have shown that mast cells and CAFs can collaborate to induce the malignant transformation of benign epithelial cells (92). Tang et al. (2022) demonstrated that CAFs secrete chemokines that recruit TAMCs in the prostate cancer stroma. Utilizing bioengineered models, researchers identified that mast cells secrete a repertoire of factors that actively remodel the ECM derived from CAFs. This dynamic reorganization of the tumor microenvironment drives a pro-tumorigenic phenotypic transition in adjacent prostate epithelial cells, thereby contributing to disease progression (93).

Once activated, TAMCs unleash a complex arsenal of cytokines and pro-inflammatory mediators that shape the tumor microenvironment, fuelling both tumor initiation and progression. In pancreatic cancer, stellate cells – progenitors of CAFs – serve as key instigators of mast cell activation, prompting their degranulation and subsequent release of cytokines. Among the secreted factors, tryptase and IL-13 play pivotal roles, stimulating stromal cell expansion and facilitating tumor growth. Beyond their pro-tumorigenic functions, TAMCs also subvert anti-tumor immunity by attracting myeloid-derived suppressor cells (MDSCs) and promoting the production of IL-17, thereby creating an immunosuppressive niche that favors malignant progression (94).

Reciprocally, the accumulation of TAMCs enhances neovascularization, increases the expression of mast cell-derived VEGF and FGF-2, and contributes to tumor aggressiveness and poor prognosis. Mast cells also produce tissue inhibitors of metalloproteinases (TIMPs), which regulate ECM degradation and facilitate the release of angiogenic factors, further supporting tumor progression (95). Evidence for mast cell–CAF cooperation, however, derives largely from *in vitro* and preclinical models and awaits confirmation in patient cohorts.

### 2.7 CAFs as regulators of dendritic cell dysfunction in the immunosuppressive tumor microenvironment

Dendritic cells (DCs) serve as indispensable antigen-presenting cells that bridge the innate and adaptive immune systems. When effectively activated, they prime T-cell responses, and their presence within the tumor microenvironment is frequently associated with improved clinical outcomes (96). DCs exist in two distinct functional states: immature and mature. A defining characteristic of mature DCs is their ability to activate naïve T lymphocytes within secondary lymphoid organs. Their maturation is triggered by disruptions in tissue homeostasis, which they detect through the recognition of pathogen-associated molecular patterns (PAMPs) or damage-associated molecular patterns (DAMPs). During this process, DCs undergo profound metabolic, cellular, and transcriptional reprogramming, enabling their migration from peripheral tissues to T-cell-rich areas of secondary lymphoid organs, where they facilitate antigen presentation and initiate adaptive immune responses (97).

Increasing evidence indicates that CAFs can modulate the differentiation and function of dendritic cells in several tumor types, ultimately subverting DC-mediated immunity and promoting tumor immune evasion. One mechanism through which CAFs impair DC differentiation is the secretion of WNT2, a factor that hinders their maturation and consequently diminishes their capacity to activate anti-tumor immune responses. Notably, therapeutic inhibition of WNT2 has been shown to enhance the presence of antigen-presenting DCs within tumors, promoting CD8+ T-cell-mediated immunity and constraining tumor progression (98). Moreover, podoplanin-expressing CAFs have been implicated in shaping specific immune signatures and facilitating DC recruitment (99).

Beyond directly suppressing DC function, CAFs contribute to the attenuation of T-cell-mediated anti-tumor immunity. The secretion of transforming growth factor-beta (TGF-β) by CAFs downregulates the expression of major histocompatibility complex class II (MHC II) molecules and co-stimulatory proteins such as CD40, CD80, and CD86 on DCs. Consequently, these cells remain in an immature state, preferentially driving the expansion of regulatory T cells (Tregs), which further suppress effector T-cell activity, reinforcing tumor immune escape. Additionally, CAFs secrete thymic stromal lymphopoietin (TSLP), conditioning myeloid DCs to favor Th2-polarised immune responses, which are associated with tumor progression (100).

The influence of CAFs extends beyond tumor promotion, as they actively modulate immune processes that shape therapeutic efficacy. Notably, in several tumor models they induce a tolerogenic phenotype in DCs, characterized by the suppression of activation markers and functional signatures, ultimately impairing their antigen-presenting capacity. However, certain radiotherapy regimens have demonstrated the potential to counteract CAF-mediated immunosuppression, thereby restoring DC functionality (101).

Furthermore, CAF-derived cytokines within the tumor microenvironment can condition DCs to adopt an immune-tolerant state, rendering them ineffective in initiating robust anti-tumor responses. This, in turn, contributes to the evasion of immune surveillance and the persistence of tumor growth (102).

## 3 Therapeutic targeting of CAFs-innate immune cells crosstalk: translational perspectives

The critical role of CAFs in orchestrating an immunosuppressive TME has motivated the development of diverse therapeutic strategies aimed at disrupting their crosstalk with innate and adaptive immune cells (12). These approaches can be broadly categorized into direct CAF depletion, functional normalization, and pathway-specific inhibition of paracrine signaling (103).

Fibroblast activation protein (FAP) has emerged as a particularly attractive target given its selective expression on pro-tumorigenic CAFs in over 90% of epithelial cancers (104). FAP-targeted strategies include antibody-drug conjugates, CAR-T cell therapies, and radiolabeled FAP inhibitors for imaging and radionuclide therapy (105). Preclinical studies consistently demonstrate that FAP-directed interventions disrupt tumor–stroma crosstalk, enhance immune-cell infiltration, and improve responses to immune checkpoint blockade. However, clinical translation has been more limited, as early studies of the anti-FAP monoclonal antibody sibrotuzumab showed minimal objective clinical responses, indicating that depletion of FAP^+^ CAFs alone is insufficient for durable tumor control (106).

The CXCL12–CXCR4 axis represents another major regulator of CAF-mediated immunosuppression. CAF-derived CXCL12 recruits immunosuppressive myeloid cells and contributes to T-cell exclusion by establishing a stromal barrier. Although CXCR4 antagonists such as plerixafor effectively restore CD8^+^ T-cell infiltration and reduce myeloid-cell recruitment in murine models, clinical studies have thus far demonstrated immune modulation without consistent long-term improvements in patient outcomes, supporting their use primarily within combination therapeutic strategies (107).

TGF-β-activated myCAFs are major architects of fibrotic immune exclusion. In experimental models, TGF-β inhibition reduces stromal fibrosis, restores cytotoxic lymphocyte infiltration, and enhances the efficacy of immune checkpoint blockade (108). Nevertheless, systemic TGF-β inhibition has demonstrated important clinical limitations due to dose-dependent toxicity and the pleiotropic physiological functions of this pathway, prompting increasing interest in selectively targeting downstream signaling mechanisms (109). Similarly, CAF-derived IL-6 promotes STAT3 activation, macrophage polarization, MDSC expansion, and therapeutic resistance. While inhibition of the IL-6/STAT3 axis has shown robust immunomodulatory activity in preclinical studies, clinical evaluation of IL-6 inhibitors, including tocilizumab-based combinations, remains investigational and has not yet demonstrated definitive survival benefits in solid tumors (110, 111).

An alternative strategy involves functional normalization rather than elimination of CAFs. Agents such as all-trans retinoic acid and vitamin D analogs convert activated CAFs toward a quiescent phenotype, reducing desmoplasia and improving immune-cell penetration (112). Despite encouraging biological rationale and promising preclinical efficacy, CAF-reprogramming strategies still lack robust clinical validation, and predictive biomarkers capable of identifying patients most likely to benefit remain an important unmet need (113).

Ultimately, the heterogeneity and plasticity of CAFs underscore the importance of rational combination strategies and patient stratification. Dual blockade of TGF-β and PD-L1 has shown particular promise in immune-excluded tumors, reshaping the TME by reducing stromal fibrosis and promoting CD8+ T-cell and NK cell infiltration (114). Similarly, combining FAP-targeted radionuclide therapy with immune checkpoint inhibitors represents an emerging frontier for converting “cold” tumors into “hot” phenotypes (115, 116). Biomarker-driven approaches that incorporate CAF subtype signatures are likely to be essential for identifying patients most likely to benefit from CAF-directed therapies (18, 117).

Collectively, these observations indicate that the greatest translational potential lies not in indiscriminate CAF depletion but in biomarker-guided targeting of specific CAF subsets combined with immunotherapy, thereby accounting for the biological heterogeneity of the stromal compartment.

## 4 Conclusion

In summary, the TME constitutes an intricate and dynamic ecosystem, where CAFs serve as central architects of immune modulation, fostering conditions that favor tumor progression. This review highlights the intricate interplay between CAFs and key immune cell populations, including TAMs, TANs, NK cells, TAMCs and DCs. Through the secretion of an array of cytokines, chemokines, and growth factors such as IL-6, TGF-β, and CXCL12, CAFs orchestrate the recruitment, differentiation, and functional reprogramming of these immune cells, steering them toward immunosuppressive and tumor-promoting phenotypes. These multifaceted interactions not only facilitate immune evasion but also drive angiogenesis and metastatic dissemination, reinforcing the suppressive nature of the TME through reciprocal regulatory circuits.

Despite these challenges, disrupting the CAF-mediated immune suppression presents a compelling avenue for therapeutic intervention. Strategies aimed at inhibiting pivotal signaling pathways – such as WNT2, TGF-β, or IL-6 – or reprogramming immune cells to adopt anti-tumor phenotypes hold promise for reinvigorating anti-cancer immunity. Future research should prioritize the dissection of the precise molecular mechanisms underpinning these CAF-immune cell interactions and explore novel approaches to dismantle the pro-tumorigenic network they sustain. Unraveling these complexities will be instrumental in the development of innovative therapeutic strategies capable of reshaping the TME and enhancing the efficacy of cancer treatments.
